# Insights into multimodal imaging classification of ADHD

**DOI:** 10.3389/fnsys.2012.00059

**Published:** 2012-08-16

**Authors:** John B. Colby, Jeffrey D. Rudie, Jesse A. Brown, Pamela K. Douglas, Mark S. Cohen, Zarrar Shehzad

**Affiliations:** ^1^Department of Neurology, University of California Los AngelesLos Angeles, CA, USA; ^2^Center for Cognitive Neuroscience, University of California Los AngelesLos Angeles, CA, USA; ^3^Department of Psychology, Yale UniversityNew Haven, CT, USA

**Keywords:** attention deficit hyperactivity disorder, ADHD-200, machine learning, classification, feature selection, fMRI, graph theory

## Abstract

Attention deficit hyperactivity disorder (ADHD) currently is diagnosed in children by clinicians via subjective ADHD-specific behavioral instruments and by reports from the parents and teachers. Considering its high prevalence and large economic and societal costs, a quantitative tool that aids in diagnosis by characterizing underlying neurobiology would be extremely valuable. This provided motivation for the ADHD-200 machine learning (ML) competition, a multisite collaborative effort to investigate imaging classifiers for ADHD. Here we present our ML approach, which used structural and functional magnetic resonance imaging data, combined with demographic information, to predict diagnostic status of individuals with ADHD from typically developing (TD) children across eight different research sites. Structural features included quantitative metrics from 113 cortical and non-cortical regions. Functional features included Pearson correlation functional connectivity matrices, nodal and global graph theoretical measures, nodal power spectra, voxelwise global connectivity, and voxelwise regional homogeneity. We performed feature ranking for each site and modality using the multiple support vector machine recursive feature elimination (SVM-RFE) algorithm, and feature subset selection by optimizing the expected generalization performance of a radial basis function kernel SVM (RBF-SVM) trained across a range of the top features. Site-specific RBF-SVMs using these optimal feature sets from each imaging modality were used to predict the class labels of an independent hold-out test set. A voting approach was used to combine these multiple predictions and assign final class labels. With this methodology we were able to predict diagnosis of ADHD with 55% accuracy (versus a 39% chance level in this sample), 33% sensitivity, and 80% specificity. This approach also allowed us to evaluate predictive structural and functional features giving insight into abnormal brain circuitry in ADHD.

## Introduction

Attention deficit hyperactivity disorder (ADHD) is among the most common child-onset neurodevelopmental disorders in the world, with an estimated childhood prevalence of 5–10% (Wolraich et al., 1996; Swanson et al., 1998), and an estimated cost in the tens of billions of dollars per year (Pelham et al., 2007) in addition to its large personal costs. Its broad spectrum of clinical features affects cognitive, emotional, and motor processes (Cortese, 2012), and clinical diagnosis typically is based on integration of parent/teacher reports and assessment of ADHD symptoms along a standardized scale (Goldman et al., 1998; Brown et al., 2001; Power et al., 2001). ADHD diagnoses can be further categorized into several different subtypes, including persistent inattention (ADHD-I), hyperactivity-impulsivity (ADHD-H), or a combination of both (ADHD-C). Like many Axis I disorders, diagnosis of ADHD hinges also on the degree to which these impairments actually interfere with daily life at school, home, and/or work (American Psychiatric Association, 2000). Medical treatment includes pharmaceutical, behavioral therapy, and/or educational interventions (Wolraich et al., 2011).

Investigations into the neurobiological basis of ADHD have found that it is highly heritable (60–75%) (Nyman et al., 2007; Faraone and Mick, 2010) and that it involves dopaminergic pathways in both the disease manifestation and the response to pharmaceutical treatment (Froehlich et al., 2011). This is consistent with observations that ADHD subjects have altered levels of dopamine (DA) transporter densities in striatal regions lateralized to the right hemisphere (McGough, 2012). Still, a clear link between genes and the heterogeneous clinical features of ADHD remains elusive. Like many behaviorally-diagnosed neurodevelopmental disorders, it is likely that multiple factors influencing several neural pathways can all lead to the ADHD phenotype (Archer et al., 2011). Therefore, it is possible that an improved understanding of the neural underpinnings of the disease may allow us to better appreciate its variation among individuals, and ultimately lead to better-targeted individual therapies.

Toward this aim, the ADHD-200 global competition challenged the neuroscientific and data mining communities to develop pattern classification methods to predict ADHD diagnosis based on a combination of demographic information, structural MRI, and resting-state functional connectivity MRI (rs-fcMRI) measurements. The data for this competition were collected as part of the Functional Connectomes Project (FCP) and the International Neuroimaging Data sharing Initiative [INDI; (Biswal et al., 2010)] as part of a push for accelerated sharing of data and analytic resources among imaging community members (Milham, 2012). The ADHD-200 initiative included the public release of neuroimaging and demographic information for nearly one thousand children and adolescents, some of whom had ADHD diagnoses, and some of whom were typically developing (TD) (Table 1). Data were included from eight participating sites, including Brown University, the Kennedy Krieger Institute at Johns Hopkins University (KKI), the NeuroIMAGE collaboration in the Netherlands (NI), New York University (NYU), Oregon Health and Science University (OHSU), Peking University, University of Pittsburgh (Pitt), and Washington University in St. Louis (WashU).

**Table 1:** **Number of subjects in training set data, by site and diagnosis**.

	**TD**	**ADHD-C**	**ADHD-H**	**ADHD-I**	**Sum**
Peking	116	29	0	49	194
KKI	61	16	1	5	83
NI	23	18	6	1	48
NYU	99	77	2	44	222
OHSU	42	23	2	12	79
Pitt	89	0	0	0	89
WashU	61	0	0	0	61
Sum	491	163	11	111	776

Site abbreviations: Peking University (Peking), Kennedy Krieger Institute (KKI), NeuroIMAGE (NI), New York University (NYU), Oregon Health and Science University (OHSU), University of Pittsburgh (Pitt), Washington University in St. Louis (WashU).

A major goal of neuroimaging research is to develop individualized measures that aid in the diagnosis and treatment of neuropsychiatric disorders. However, the robustness of differences at the individual subject level is not well established since most studies typically report group level differences and do not use independent replication samples. When neuroimaging data are analyzed under the framework of machine learning (ML), the focus is to develop a classifier that can be used to predict disease status for individual subjects. The top features contributing to the classifier outcome can also be examined to better understand alterations in the brain circuits of individuals with a given disorder (O'Toole et al., 2007; Ecker et al., 2010; Hanke et al., 2010). Over the past several years, classification methods have been increasingly applied to neuroimaging data to identify individuals with Alzheimer's disease (Klöppel et al., 2008; Supekar et al., 2008), schizophrenia (Davatzikos et al., 2005), and autism (Ecker et al., 2010; Ingalhalikar et al., 2011) from healthy controls. The approaches taken, including data type (fMRI, functional connectivity fMRI, diffusion tensor MRI, structural MRI) and methods (feature selection and type of classifier) have varied considerably. Although disease classification of neuroimaging data has shown considerable promise, most studies have used relatively small sample sizes without replication samples (Linden, 2012). Since classifiers can perform better with larger training samples, pooling data across multiple sites is an important direction for the field and one that is being taken by the ADHD-200 global competition.

In the present paper, we (1) briefly review some of the key structural and functional neuroimaging findings that are thought to differentiate ADHD from TD individuals, (2) present the ML approach that we applied to the ADHD-200 competition, (3) explore which feature modalities and brain regions proved to be the most useful for classification, and (4) reflect on important areas of broader insight and future directions that can be drawn from the ADHD-200 initiative, due to its unique position as the largest neuroimaging ML effort to date.

Converging evidence from both structural and functional neuroimaging studies consistently have demonstrated that individuals with ADHD have alterations in fronto-striatal circuitry (Emond et al., 2009). For example, structural studies using voxel-based morphometry (VBM) have reported decreased gray matter volume in the right inferior frontal gyrus in ADHD subjects (Depue et al., 2010). Recent meta-analyses of structural differences also report less gray matter in the right hemisphere in ADHD samples, specifically in basal ganglia regions including the caudate, putamen, and globus pallidus (Ellison-Wright et al., 2008). Thinner cortex has also been observed in ADHD subjects (Narr et al., 2009), particularly in right frontal regions (Qiu et al., 2011), and correlates with disease severity (Almeida et al., 2010). Nonetheless, the results from quantitative structural studies have varied (Castellanos and Proal, 2009), as morphological alterations appear to resolve to some extent over the course of development (Larisch et al., 2006), and after treatment with stimulants that enhance DA signaling (Shaw et al., 2009).

Evidence from functional MRI studies has generally paralleled that of structural neuroimaging (Liston et al., 2011). For example, task-based fMRI studies have found hypoactivity in frontal and striatal regions characteristic of ADHD (Zametkin et al., 1990; Christakou et al., 2012). Functional changes have also been observed in cerebellar and parietal areas (Cherkasova and Hechtman, 2009). Functional neuroimaging studies using rs-fcMRI have implicated alterations in functional connectivity between multiple brain regions in ADHD (Castellanos et al., 2009; Fair et al., 2010; Bush, 2011). In particular, the brain's default mode network (DMN) has proven useful in understanding the pathology of ADHD (Zang et al., 2007) and a number of other mental disorders (Broyd et al., 2009). The DMN is one of several “intrinsic” or “resting-state” networks that are composed of distributed sets of brain regions (“nodes”) that vary coherently at low frequency (Fox and Raichle, 2007; Buckner et al., 2008). The DMN is generally activated when an individual is not focusing on external stimuli, and, during goal-oriented tasks, these low frequency fluctuations typically are attenuated (Raichle, 2001). One theory on the neurobiological basis of ADHD is that these individuals may have diminished ability to inhibit this default processing (Fassbender et al., 2009), and thus consequently they have a diminished ability to focus on external goal-oriented tasks. Rs-fcMRI studies in ADHD have revealed diminished coherence between the prefrontal cortex (PFC) and posterior cingulate cortex (pCC; a major integration node of the DMN) in individuals with ADHD (Castellanos et al., 2008; Fair et al., 2010). An overall decreased network homogeneity, particularly with respect to precuneus functional connectivity, has also been reported in resting state data from ADHD children (Uddin et al., 2008). Finally, complex network modeling approaches [i.e., graph theory; (Bullmore and Sporns, 2009)], which characterizes the brain as a set of “nodes” (brain regions) and “edges” (connections between nodes), have reported differences in local and global functional network properties in ADHD (Wang et al., 2009).

Taken together, this evidence suggests that measures of structural brain morphology and rs-fcMRI may be useful in differentiating ADHD from TD. However, given the heterogeneity of the findings and methods, as well as small sample sizes used in previous studies, it is unclear which set of features or methods might be the most useful for classification.

## Methods

### Overview

Our method in brief is as follows: first, we quantified neuroimaging features from structural and functional data from all subjects. Feature ranking for each site and imaging modality was then performed using the linear support vector machine recursive feature elimination (SVM-RFE) algorithm. After preliminary explorations into the variability of the feature usefulness rankings across sites, we chose to perform classification within site where possible. Optimal feature subsets were then selected for each neuroimaging feature modality and for each site. The number of top features to use was chosen based on maximizing the expected generalization performance of a radial basis function kernel SVM (RBF-SVM). These performance estimates were generated using 10-fold cross validation, which was external to the feature ranking/selection so as to remain unbiased by spurious features, as well as a standard layer of internal 10-fold cross validation to tune the model hyperparameters. Site-specific RBF-SVMs were then retrained on *all* observations in the training datasets, while using only the optimal number of top features. These were used to predict the class labels of the test dataset using features from each imaging modality independently. Lastly, simple voting was used to combine these multiple predictions and assign final class labels.

### Features

As part of the ADHD-200 competition, a training dataset was released first; it included structural and functional imaging data from 776 individuals (491 TD and 285 ADHD), their diagnostic class labels (TD or ADHD subtype), and accompanying demographic information (Table 1). Imaging data for all subjects included one or more resting-state functional MRI scans, and a high resolution T1-weighted anatomical scan. For our analysis of the resting-state fMRI data, we utilized the already preprocessed fMRI data provided by the Neuro Bureau, and made available to all users at http://neurobureau.projects.nitrc.org.

Broadly, this fMRI preprocessing procedure involved slice timing correction, motion correction, registration of the fMRI data into MNI152 standard space at 4 mm^3^ resolution, regression of nuisance parameters for WM, CSF, and motion parameters, band-pass filtering the timeseries data from 0.009–0.08 Hz, and spatial smoothing with a 6 mm full width at half maximum Gaussian filter. For details of the preprocessing and software use, see http://www.nitrc.org/plugins/mwiki/index.php/neurobureau:AthenaPipeline.

#### Demographics

**Figure 1:**
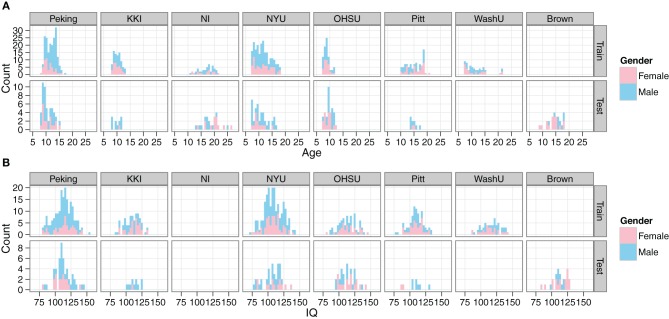
**Comparison of demographics distributions in training and test sets.** Histograms are shown for age **(A)** and IQ **(B)**. Gender is encoded by color. Plots are facetted by Site and training/test set. Site abbreviations: Peking University (Peking), Kennedy Krieger Institute (KKI), NeuroIMAGE (NI), New York University (NYU), Oregon Health and Science University (OHSU), University of Pittsburgh (Pitt), Washington University in St. Louis (WashU), Brown University (Brown).

Demographic data from the training set included age, gender, full-scale IQ, handedness, ADHD index measurements, hyperactive/impulsivity and inattentive scores, secondary diagnosis, and medication status. However, all of the ADHD-related information was withheld from the test set. In our initial explorations with the data, we created site-by-site distributions of the main remaining features (age, gender, full-scale IQ) for both the training and test data, in order to verify that they appeared to be drawn from the same populations (Figure 1).

#### Structural and morphological features

Raw T1-weighted anatomical MRI scans were processed with Freesurfer's *recon-all* processing pipeline for whole brain segmentation and parcellation (Fischl and Dale, 2000). This generates segmentations of white matter, gray matter, and subcortical volumes. A mesh model of the cortical surface is generated, which is then subdivided into different cortical regions (e.g., precentral gyrus, superior frontal gyrus, pars triangularis, etc.). For each region, the program measured the number of surface vertices, surface area, gray matter volume, average cortical thickness, cortical thickness standard deviation, cortical mean curvature, cortical Gaussian curvature, cortical folding index, and cortical curvature index. These nine measures were calculated for 34 cortical regions per hemisphere. We also calculated morphological measures from 45 non-cortical regions including subcortical regions, white matter, ventricles, and other non-gray matter entities (i.e., white matter hyperintensities); these measurements included regional volume, regional voxel intensity mean, and regional voxel intensity standard deviation. A total of twenty subjects from the training set were excluded on account of data quality/processing issues.

#### Functional neuroimaging features

***Resting state functional connectivity matrices.*** For each subject, we created rs-fcMRI matrices by calculating the Pearson pairwise correlation between BOLD time-series extracted from the Athena (Neuro Bureau) preprocessed unfiltered data, for brain regions of interest (ROIs) as defined in standard atlases. Several atlases were explored that ranged in mean ROI volume. First, we used the Harvard–Oxford (HO) atlas, an anatomical atlas based on gyral and sulcal tracing with ~100 brain regions. We also used the CC400 atlas (Craddock et al., 2011), a functionally-derived atlas with ~400 ROIs from the Athena pipeline. Time-series correlations between each of the brain regions were measured, and correlation coefficients were z-transformed in order to generate whole brain functional connectivity matrices for each subject using MATLAB (The Mathworks, Natick, MA). These connectivity matrices are square and symmetric, so the lower triangle of each was used as ML features. These matrices also were used to calculate nodal and global graph network features, as described below.

***Nodal and global graph measures.*** We used the Brain Connectivity Toolbox in MATLAB (Rubinov and Sporns, 2010) to compute weighted global and nodal graph theoretical metrics on rs-fcMRI connectivity matrices based on three atlases: the HO, CC400, and 90 functional ROIs from the Stanford FIND lab (http://findlab.stanford.edu/functional_ROIs.html). We normalized network sparsity across subjects by taking the same percentage of each subject's strongest positive connections before calculating graph theoretical metrics. Global and local and network properties were calculated at 10, 15, 20, 25, and 30 percent sparsity and averaged across these sparsity levels.

We computed eight *global* graph theoretical metrics (Rubinov and Sporns, 2010). Global metrics were *clustering coefficient* (CC) and *local efficiency*, which measure the degree to which neighbors of a node are connected to each other; *characteristic path length* (CPL), which represents the average number of edges needed to get from any node in the network to any other node in the network; *global efficiency*, which is similar to the inverse of CPL but can be computed for networks that are not fully connected, normalized CC and CPL (*gamma* and *lambda*), which are calculated as a ratio of CC or CPL to the average CC or CPL, respectively, of 100 simulated random networks with equivalent numbers of nodes and edges; *small worldness*, which is the ratio of gamma to lambda (Watts and Strogatz, 1998); and *modularity*, a measure of the degree to which the network can be subdivided into nonoverlapping subnetworks that are maximally connected within and minimally connected without. We also computed five *nodal* metrics for each node: strength (number of connections), CC, local efficiency, regional efficiency (the inverse of average path length from the node to any other node in the network), and between-ness centrality (the fraction of shortest paths in the entire network that traverse through a given node). These were calculated for each subject and compared between groups. The eight global metrics and nodal metrics for each node were used as features in classification. Nodal and global metrics were computed for both binarized and weighted networks to test which method would perform better in classification for each site.

Renderings were generated using the UCLA Multimodal Connectivity Package (http://github.com/jbrown81/umcp) and through the UCLA Multimodal Connectivity Database (http://umcd.humanconnectomeproject.org), which use the Python libraries networkX (http://networkx.lanl.gov) and matplotlib (http://matplotlib.sourceforge.net). All connectivity matrices from the CC atlas are publicly shared and available for download and analysis at http://umcd.humanconnectomeproject.org.

***Nodal power spectrum.*** For each participant, we used **R** (http://www.r-project.org) to obtain the power spectrum for each of the CC400 ROIs. We converted each ROI's time-series into the frequency domain using the Fourier transform. The power spectrum was then obtained by taking the modulus of the real and imaginary portions of the data ($\sqrt{Re^{2}+Im^{2}}$).

***Voxelwise global connectivity.*** For each participant, we obtained a measure of each voxel's global connectivity using AFNI's 3dTcorrMap (Cole and Schneider, 2007; Buckner et al., 2009). This involved two steps. First, for each voxel, we calculated the correlation between that voxel's time-series and that of every other voxel in gray matter. Second, the average was taken for each voxel's Fischer-z transformed correlation with every other gray-matter voxel.

***Voxelwise regional homogeneity.*** For each participant and each voxel, we used **R** to calculate the consistency of a voxel's time-series with its 26 spatially adjacent neighboring voxels using Kendall's W (Zang et al., 2004).

### Machine learning

Our general approach to classification included (1) feature ranking and optimal subset selection, (2) training site- and modality-specific classifiers using these optimal feature lists, (3) predicting the unknown class labels for the test set, and (4) final outcome voting to combine modalities. All analyses were performed using R, and the tools we developed are freely available at http://github.com/johncolby/SVM-RFE.

#### Feature ranking

Given the accelerating rate of data being collected across different fields such as genetics and neuroimaging, one of the key challenges is in mining these data effectively to distill large numbers of features into more useful summaries (Guyon, 2003). Therefore, when considering the vast ADHD-200 dataset, a crucial component was determining how to limit which features to include in the final classification vector. While the task of feature selection is difficult for any dataset, it becomes even more complex when classification is performed on multimodal data, where the features themselves are represented in different spaces and may vary in number over many orders of magnitude.

It is known that both redundant and extraneous features can degrade the performance of a given classifier, even with a small number of “noise” features (Kohavi and John, 1997; Farahat et al., 2011). Furthermore, when the number of features is large compared to the number of observations in the training dataset, there are a large number of ML parameters to solve. This can decrease interpretability and the capability of the model to generalize to new datasets. It is therefore useful to perform some sort of dimensionality reduction or feature selection, particularly when there are many features present (e.g., the CC400 rs-fcMRI matrix begins with 160,000 initial elements, and produces 79,800 features after duplicates and self-correlations are removed).

We chose to apply the linear SVM-RFE algorithm (Guyon et al., 2002) to obtain a ranked list of features. The decision was driven by the established theory of both SVM and RFE, and the long history and successful application of SVM-RFE to microarray-based diagnostic classification (Johannes et al., 2010; Shi et al., 2011). This is a similarly medical application, which also involves a large number of strongly correlated features. More recently, SVM-RFE has also been applied successfully in several neuroimaging applications for feature selection across functional connectivity data (De Martino et al., 2008; Craddock et al., 2009; Deshpande et al., 2010), which is even more directly relatable to the ADHD-200 challenge. SVM-RFE, as its name would suggest, works backwards from the initial full set of features and eliminates the least “useful” feature on each recursive pass. In contrast to optimization methods that can revisit locations in feature space [e.g., genetic/evolutionary algorithms (Vafaie and Imam, 1994)], in SVM-RFE once a feature is removed, it will not be reconsidered on subsequent passes of the algorithm. The criterion used to judge feature usefulness in SVM-RFE is the absolute value of the feature weight from a linear SVM fit to the dataset. Linear SVM is a linear discriminant, in that it seeks to find a *linear* combination of the features that allows for the best classification of groups. Whereas the classical Linear Discriminant Analysis (LDA) interpretation seeks to maximize the ratio of the between-class variance to the within-class variance in the standard ANOVA sense, SVM seeks a discriminant function that maximizes the distance (the “margin”) to the nearest training set observations of either class (the “support vectors”). The theory was described originally by Vapnik and Lerner (1963), and later extended to accommodate the exceedingly common situation where the classes are *not* completely separable, requiring some training examples to remain mislabeled in the solution (Cortes and Vapnik, 1995). This decision boundary ends up as a line in two dimensional feature space and as a higher dimensional hyperplane when more features are present. Because linear SVM assigns multivariate weights to all remaining features at once, it has the ability to accommodate highly correlated features, as well as potential mutual information between features that might not be very useful on their own. This approach contrasts with univariate correlation-based feature ranking, where features are ordered, for example, by conducting simple between-group *t*-tests for each (Guyon et al., 2002).

**Figure 2:**
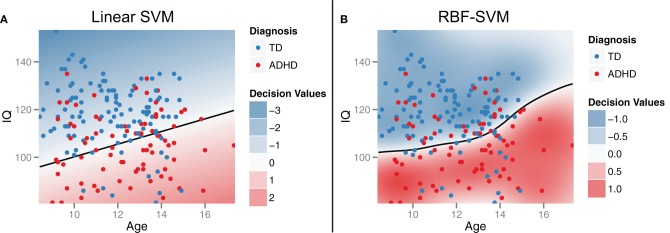
**Support vector machines (SVM).** Support vector machines identify a classification decision boundary (shown as a black line) that maximizes the margin (i.e., Euclidean distance) to the nearest training cases of either class, subject to a misclassification penalty. In linear SVM **(A)**, this boundary is a line in two dimensions and a hyperplane in higher dimensions. Kernel transformations [for example, using a radial basis function (RBF)] can be applied to allow for a non-linear decision boundary in the original feature space **(B)**. Example data were drawn from the male participants at the Peking site.

To demonstrate the idea of linear SVM-RFE, consider the simplified 2-dimensional, 2-class, case of distinguishing ADHD from TD subjects from the male Peking subjects based on age and IQ alone (Figure 2A). This corresponds to the bottom-left panel in Figure 6. If we plot the results of the linear SVM fit, we can see that the decision boundary cuts more along the IQ axis. This means that the IQ feature has a higher weight than age, and that age would be dropped first in the recursive elimination algorithm.

We also chose to apply two modifications to the original SVM-RFE algorithm: (1) Due in part to its multivariate nature, as well as simple sampling variability, some of the feature rankings output from the SVM-RFE algorithm can be unstable (Craddock et al., 2009). Because of the large number of training cases in this study however, resampling methods provide a simple route to improving the stability of these rankings. We chose to use the *multiple* SVM-RFE (mSVM-RFE) extension described by Duan et al. (2005), which imposes a resampling layer on each recursion pass such that the weights used for feature ranking/dropping are stabilized by averaging across results for multiple subsamples. (2) For computational considerations, we chose to drop half of the features on each pass until the remaining number of features dropped below 5000, at which point the algorithm switched to a one-by-one mode to give the most accurate rankings of the top features.

#### Main classifier

Based on initial exploration of the demographic data, we were able to identify early on that these features would be highly useful compared to the types of effects we expected to see across the imaging feature set. Therefore, since we knew these features would form the core of our classifier, we compared different classification approaches on these features alone as a foundation for building up the rest of our classifier. We investigated several common ML approaches, including: linear SVM, SVM with a RBF-SVM, decision stumps as a base classifier in adaboost, random forests, and C4.5 decision trees. RBF-SVM gave the best expected generalization performance (See Section “Expected classifier performance”), so we continued with that approach as our main classifier. RBF-SVM is similar to the linear SVM approach previously discussed as part of the feature ranking algorithm, but employs a kernel transformation to allow for a non-linear decision boundary in the original feature space (Figure 2B). The radius of the kernel parameter and the soft margin misclassification penalty were both tuned using standard methods for nested 10-fold cross validation and grid search. This maximizes accuracy and lowers the chance of overfitting.

Because of the large and site-specific skews in ADHD subtype prevalence (See results in Section “Diagnosis and site” and discussion of these site-specific effects in Sections “Site” and “Classification across sites”), we decided to focus our ML efforts on the 2-class problem of TD vs. ADHD, and assign ADHD subtypes in a site-specific manner based on the most common subtype present in the training data.

#### Optimal feature subset selection

Once we obtained ranked lists of the features for each imaging modality using SVM-RFE, the next step was to select the optimal subsets of these top features for use in our final classifier. This is an important step in optimizing many types of ML classifiers, as it is desirable to keep enough features to capture the most important aspects of the data with respect to classification, but not so many as to lead to overfitting and poor generalization performance.

**Figure 3:**
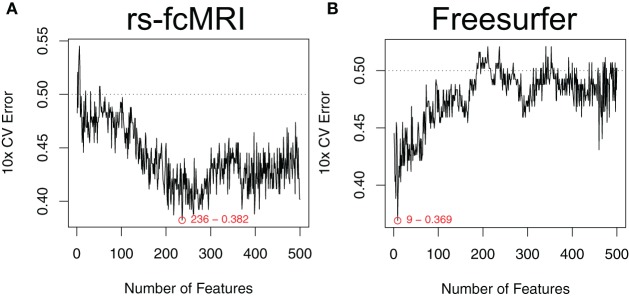
**Optimal feature subset selection.** Feature subsets were chosen to optimize the expected generalization performance of the classifier. Performance was estimated using a 10-fold cross validation procedure that was external to the feature ranking (Ambroise and McLachlan, 2002). This process was repeated over a range of the top features. Example performance vs. number-of-features curves are shown for raw rs-fcMRI connectivity matrix data **(A)** and Freesurfer morphological data **(B)**. Example data were drawn from the NYU site. Plots are annotated with the optimal number of features and the associated expected generalization performance.

Estimated generalization performance was determined using a layer of 10-fold cross validation. Within each fold, the classification accuracy on the hold-out samples was repeatedly gauged, while varying the number of the top features used as input. Averaging across all 10 folds allowed us to generate plots of generalization performance vs. number of features (Figure 3). The minimum along the curve was selected as the optimal subset of features to use in our final classifier. Importantly, this estimation of generalization performance and 10-fold cross validation was *external* to the feature ranking step. In other words, the features were ranked 10 times, each time independent of the hold-out samples for that given fold. This ensures that the estimated generalization performance is unbiased by spurious features that might nicely explain the training class labels but don't generalize to the population (Ambroise and McLachlan, 2002).

#### Patterns among top features

The feature selection stage returns a ranked list of all the features for each site and imaging modality, as well as the number of top features that were expected to give the best generalization performance. These were used primarily to choose which features should be included in the final site-specific classifiers, as discussed next in Section “Site-specific classifiers”. Additionally, however, patterns among the most useful features can be investigated directly, as a multivariate alternative to traditional voxelwise univariate hypothesis testing. In this manuscript we focus on the NYU site as a representative example, and generate back-projected plots of feature rankings in the space of the original imaging modalities (i.e., color coded onto the brain surface for the morphological features, and as graph theoretical, regional homogeneity, global connectivity, and power spectra visualizations for the rs-fcMRI analysis).

#### Site-specific classifiers

**Figure 4:**
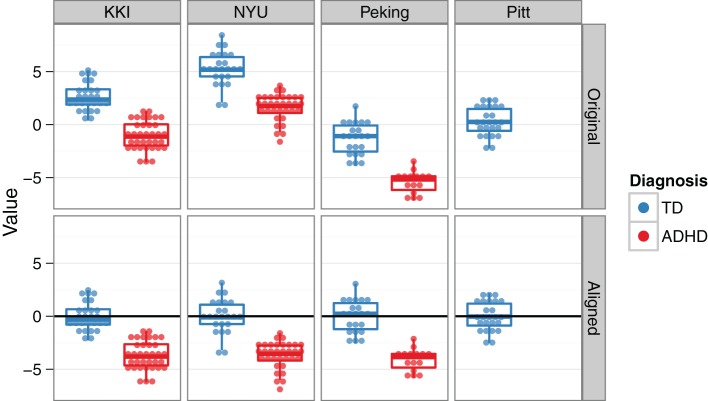
**Across-site alignment.** For sites without complete training data (e.g., Pittsburgh), sites were aligned by their available subgroups (here, TD) and then an across-group classifier was trained. This schematic uses artificial data to demonstrate the procedure.

To address the multisite aspect of the ADHD-200 dataset, we employed a collection of site-specific classifiers, rather than a single classifier with site as a feature. This allowed us to tailor feature selection to each site, while also accommodating the unique site-specific aspects of the data set (See Sections “Demographics” and “Classification across sites”). Site-specific classification was also chosen based on initial findings of heterogeneity of top features and above chance prediction accuracies between different sites. For example, because of differences in study inclusion criteria or varying T1-weighted scan qualities and acquisition parameters, the morphological features might be very useful at one site, but not at another. Additionally, this approach is able to handle missing features (e.g., IQ is not reported from the NI site), since the classifier for that site can simply be trained without them. For the Pitt site, although there were no ADHD subjects in the training data, the available TD subjects were used to align TD feature means across sites, and thus allowed us to tap into the discriminating aspects of the data from the other sites (Figure 4). For the Brown site, which was the most challenging because it lacked *any* training data, a similar across-site classifier was used. However, its feature-wise bias adjustment was cruder than the Pitt site's, because the unknown class labels required that the alignment be based on both ADHD and TD subjects together. The WashU site was excluded altogether from our final classification approach, since it was not part of the test set, and we reasoned that any explanatory benefit from including the training data would be outweighed by the simultaneous increase in the between-site variance.

#### Combining modalities

Feature ranking and optimal feature subset selection were performed independently for each imaging modality. We chose this approach so that the tens of thousands of fMRI features would not swamp the much fewer number of morphological features, and so that we could choose the most effective processing options (e.g., 200 nodes, vs. 400 nodes for extracting graph theory metrics from the fMRI time-series data) among different preprocessing runs for the same modality. For each site, a set of RBF-SVMs were then trained: once for each imaging modality, once for the demographics alone, and once with all the top features from all modalities together. These were used to generate a list of class predictions for each test set subject.

**Figure 5:**
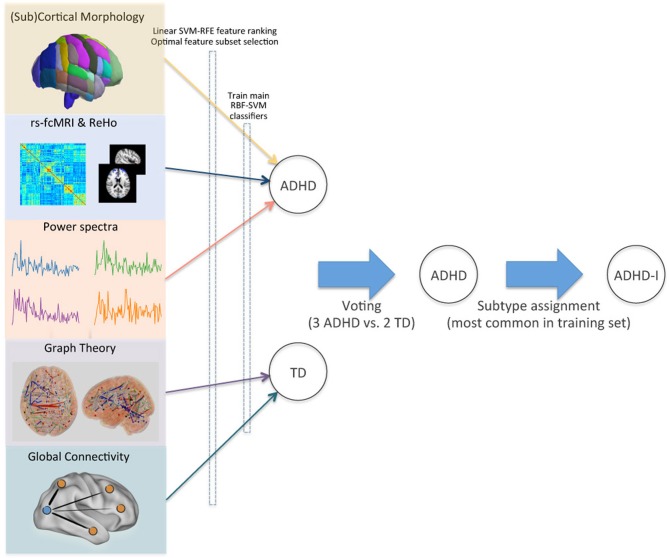
**Voting method for combining modalities.** Optimal feature subsets were derived for each site and imaging modality, and used to train a set of RBF-SVM classifiers. Class predictions from these modality-specific classifiers were then used as inputs for final voting. ADHD subtypes were assigned based on the site-specific pretest probabilities in the training set. This schematic demonstrates the class assignment procedure for a single example subject.

To assign the final class labels, we combined the individual class predictions from the different feature sets with a higher level voting procedure (Figure 5). For each test subject in the ADHD-200 competition, the most common class in the set of predictions determined the final class label.

## Results

### Demographics

#### Diagnosis and site

The prevalence of ADHD across the *entire* training set was 37%. Ignoring the two sites without any ADHD subjects in their training data, the prevalence was 46%. We also observed prominent differences in ADHD prevalence and ADHD subtype ratios between sites (Table 1). The ADHD-H subtype was the lowest represented among the three subtypes, and prior information from the ADHD-200 contest indicated that no subjects with this diagnosis would be included in the test set. We therefore excluded these subjects, and constrained our diagnostic predictions to three classes. Of the remaining subjects, the highest prevalence of ADHD was 55% at the NYU site, and the lowest was 27% at the KKI site. For the two main ADHD subtypes, ratios varied widely between sites—from 18:1 ADHD-C:ADHD-I at the NI site to nearly 3:5 at the Peking site.

#### Gender

In aggregate across the training set, ADHD diagnoses were far less common in females than in males. Ignoring the sites with no ADHD subjects, the prevalence of ADHD in females was 27%, but in males it was 54% (Table 2). These numbers also varied strongly by site. For example, at Peking, only 13% of female training subjects were ADHD, while at NYU 64% of males were ADHD.

**Table 2:** **Number of subjects by site, diagnosis, and gender**.

	**TD**	**ADHD-C**	**ADHD-I**	**Sum**
**(A) FEMALES**				
Peking	45 (86.5)	0 (0.0)	7 (13.5)	52 (100.0)
KKI	27 (73.0)	9 (24.3)	1 (2.7)	37 (100.0)
NI	12 (75.0)	4 (25.0)	0 (0.0)	16 (100.0)
NYU	52 (65.8)	12 (15.2)	15 (19.0)	79 (100.0)
OHSU	24 (70.6)	4 (11.8)	6 (17.6)	34 (100.0)
Sum	160 (73.4)	29 (13.3)	29 (13.3)	218 (100.0)
**(B) MALES**				
Peking	71 (50.0)	29 (20.4)	42 (29.6)	142 (100.0)
KKI	34 (75.6)	7 (15.6)	4 (8.9)	45 (100.0)
NI	11 (42.3)	14 (53.8)	1 (3.8)	26 (100.0)
NYU	47 (33.6)	64 (45.7)	29 (20.7)	140 (100.0)
OHSU	18 (41.9)	19 (44.2)	6 (14.0)	43 (100.0)
Sum	181 (45.7)	133 (33.6)	82 (20.7)	396 (100.0)

Females (A) and males (B) are shown separately. Percentages of row totals are given in parentheses.

#### Age and IQ

**Figure 6:**
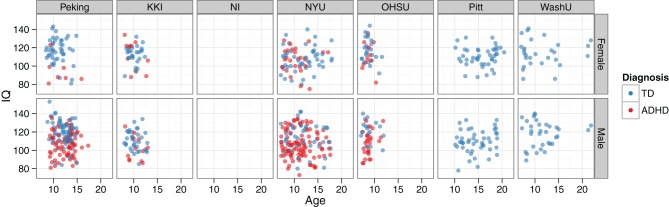
**IQ vs. age, by diagnosis, site, and gender.** Demographics data are plotted for the training set. The NeuroIMAGE site had no IQ data, and the Pittsburgh and Washington University sites only had TD cases. The bottom left panel corresponds to the example data used in Figure 2.

Beyond looking at prevalence rates across sites and genders, the two main continuous-valued demographic features made available were age and a full-scale IQ score. The relationships between age, IQ, and diagnosis—together with how these vary by site and gender—can all be visualized simultaneously (Figure 6).

#### Expected classifier performance

As a baseline reference for building our imaging-based classifier, we explored the generalization performance we could expect from training a classifier on only the demographics features. Age, gender, site, and IQ features were included from the four sites with complete data (Peking, KKI, NYU, and OHSU), and used to train an RBF-SVM classifier (e.g., Figure 2B). Using these features alone, predicted 3-class generalization accuracy was 62.7%, sensitivity was 30%, and specificity was 92%. Taking into account the fact that greater emphasis was placed on correct TD diagnoses, this would correspond to achieving 65% of the total possible points in the competition.

### Feature selection

We investigated patterns among the top features to see whether they localized to regions previously reported to be affected in individuals with ADHD. Note: These analyses are only showing data from the NYU site, which was chosen as a representative example.

**Figure 7:**
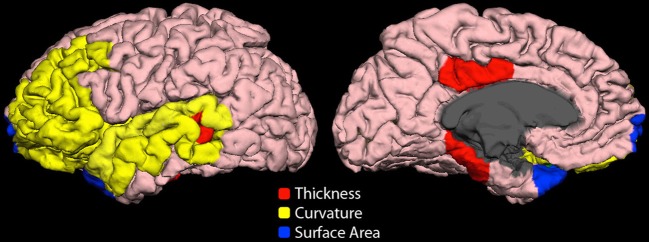
**Cortical features (NYU site).** Twelve cortical morphological measures were optimally discriminative of class, including cortical thickness (posterior cingulate, parahippocampal, bank of superior temporal sulcus), cortical curvature (superior temporal, lateral orbitofrontal, rostral middle frontal, middle temporal, transverse temporal, pars triangularis, and insula), and cortical surface area (frontal pole and temporal pole).

Highly ranked cortical structural features used to generate output votes are listed in Table 3. For the NYU cohort, the optimal feature set included 13 cortical morphological measures, including cortical thickness, curvature, and surface area (Figure 7).

**Table 3:** **Top-ranked Freesurfer cortical features (NYU site)**.

**Ranking**	**Cortical Region**	**Measure**
1	Posterior cingulate	ThickStd
2	Bank of superior temporal sulcus	ThickAvg
3	Superior temporal	MeanCurv
4	Frontal pole	SurfArea
5	Lateral orbitofrontal	MeanCurv
6	Rostral middle frontal	GausCurv
7	Parahippocampal	ThickAvg
8	Parahippocampal	ThickStd
9	Temporal pole	SurfArea
10	Middle temporal	CurvInd
11	Transverse temporal	GausCurv
12	Pars triangularis	CurvInd

Regions from the Freesurfer structural analysis for the NYU site that were included in the classification. ThickStd: cortical region thickness standard deviation, ThickAvg: cortical region thickness average, SurfArea: cortical region surface area, MeanCurv: cortical region mean curvature, GausCurv: cortical region Gaussian curvature, CurvInd: cortical region curvature index.

**Figure 8:**
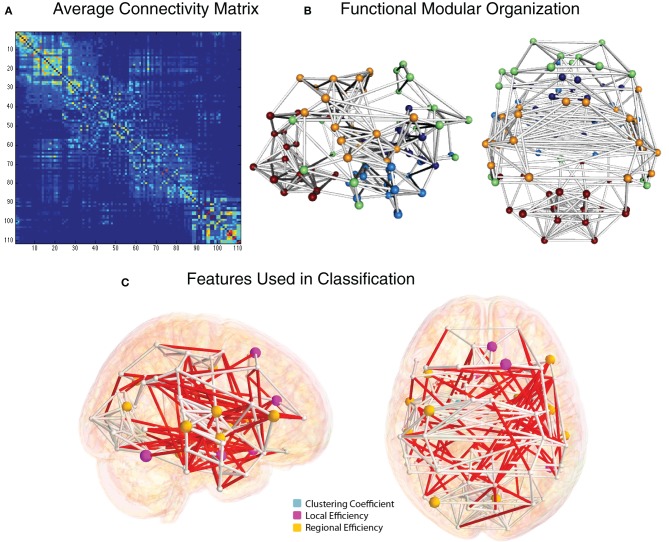
**Functional connectivity and graph theory features (NYU site). (A)** Average NYU functional connectivity matrix using 111 cortical and subcortical regions from the Harvard Oxford atlas reorganized by modular organization as detected by the Louvain modularity algorithm. **(B)** Group-averaged functional connections from the NYU cohort, shown from right and top views. Spheres correspond to the center of mass for the 111 and white cylinders correspond to connections in the top 2% of functional connectivity strength, based on Pearson correlation. **(C)** Nodes whose graph theory-based measures were used to classify ADHD vs. TD are shown in light blue (clustering coefficient), magenta (local efficiency), and yellow (regional efficiency). Edges whose connection weights were used in the classification are shown in red.

The group-average functional connectivity matrix from the HO atlas from the NYU cohort is shown in Figure 8A. This same matrix is rendered as a 3D network in Figure 8B. In this representation, nodes are shown as spheres at the center of mass of each ROI, with color corresponding to module membership based on the Louvain modularity algorithm (Blondel et al., 2008). The strongest 1% of connections are shown as bars connecting nodes. Specific edges colored in red and graph theory-based nodal features that were included in the classification vector for the NYU site are shown in Figure 8C.

**Figure 9:**
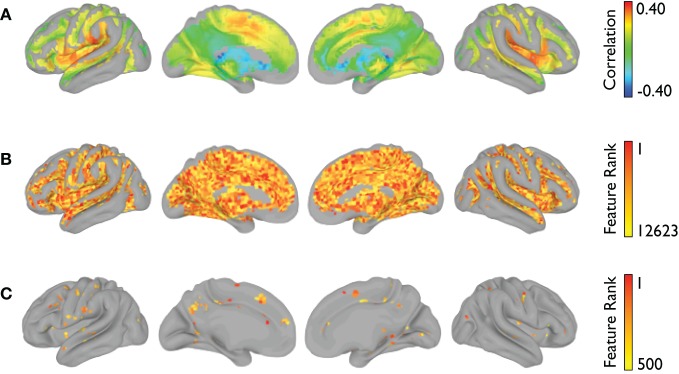
**Global connectivity feature rankings (NYU site).** For each voxel present in all participants (across all sites), **(A)** the group-averaged correlation with every other voxel, **(B)** the average feature rankings from the within-site classification, and **(C)** the top 500 features from the within-site classification are shown.

**Figure 10:**
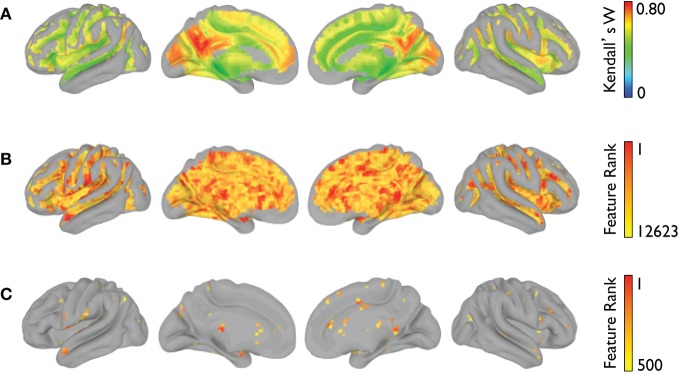
**Regional homogeneity feature rankings (NYU site).** For each voxel present in all participants (across all sites), **(A)** the group-averaged consistency of each voxel with its (26) nearest neighbors, **(B)** the average feature rankings from the within-site classification, and **(C)** the top 500 features from the within-site classification are shown.

The voxelwise group-average global connectivity measure is shown in Figure 9A. It is accompanied by the feature rankings for all voxels (Figure 9B) and the top 500 voxels (Figure 9C). Similarly, the voxelwise group-average regional homogeneity measure is shown in Figure 10A. It is also accompanied by the feature rankings for all voxels (Figure 10B) and the top 500 voxels (Figure 10C).

**Figure 11:**
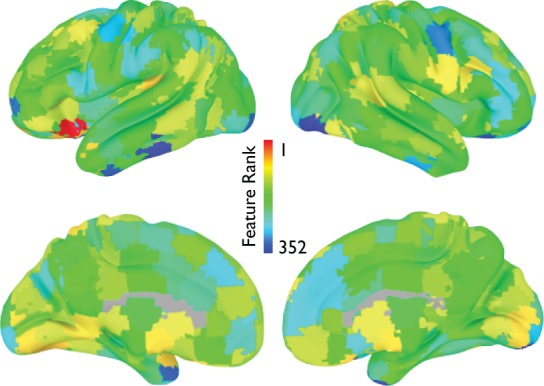
**Power spectrum feature rankings (NYU site).** For each ROI in the CC400 atlas, the average feature rankings across each frequency bin of the power-spectrum are displayed. Warmer colors indicate more useful features.

The average feature rankings across the power spectra for each of the CC400 ROIs are shown in Figure 11. Areas with prominent feature rankings include ROIs in the left IFG/insula, left DLPFC, and subcortical areas.

### ADHD-200 competition results

The performance of our ML approach was judged on an independent hold-out test set of 197 individuals as part of the ADHD-200 Global Competition. Classifier performance metrics were reported to us by the competition organizers (http://fcon_1000.projects.nitrc.org/indi/adhd200/results.html). Considering all three classes (TD, ADHD-I, ADHD-C), our overall accuracy was 55%. The chance level associated with blindly guessing one of the three diagnostic classes was 33%. The chance level associated with hierarchical coin-flipping (i.e., flip once to guess ADHD vs. TD, and again for those ADHD in order to guess subtype) was reported by the organizers to be 39%. The chance level associated with predicting the entire test set to be members of the most common training set class (TD) was 55%. Considering only the two main classes (TD, ADHD), the sensitivity (i.e., percent correct ADHD) of our approach was 33%, and the specificity (i.e., percent correct TD) was 79%. We correctly predicted the ADHD subtype in 76% of those subjects correctly classified as ADHD.

This corresponded to receiving 110.5 out of a possible 195 points, 4th place out of 21 total entries in the competition, and 3rd place among the teams that used the imaging data for classification.

### Classifier statistics by site and modality

After the ADHD-200 competition was finished, the true class labels for the test set were released to the community. This allowed us to perform *post-hoc* analyses to determine which imaging modalities were the most useful across sites for the classification of ADHD. Keeping in line with the official results, 3-class overall accuracy, 2-class sensitivity and specificity, and ADHD subtype accuracy were calculated for each combination of feature modality and site (Table 4). However, it should be noted that these values are not quantitatively comparable to the official competition results because not all of the test set labels have been released. Of the combinations investigated, the best performance was achieved using the combination of imaging and demographics features that we actually implemented during the competition. Out of the individual imaging modalities, however, the fMRI power spectrum features appear to have been the most useful. Based on feature ranking and optimal feature subset selection on the training set, these features were predicted to be useful at 4 out of the 5 sites with available data. On the test set, these features delivered an average 57% accuracy, which was the highest of the common imaging modalities. For these power spectrum features, their sensitivity of 25% for detecting ADHD was toward the bottom of the list of modalities, their specificity of 83% was near the top, and their ADHD subtype accuracy of 75% was near the middle.

**Table 4:** **Classifier performance on the test dataset, by site and imaging modality**.

**Modality**	**Metric**	**Site**					
		**KKI**	**NI**	**NYU**	**OHSU**	**Peking**	**All**
Combined	Accuracy	0.73	0.68	0.37	0.76	0.57	0.59
	Sensitivity	0	0.45	0.34	0	0.25	0.24
	Specificity	1	0.86	0.58	0.93	0.96	0.85
	Subtype	–	1	0.8	–	0.5	0.71
Combined (no IQ)	Accuracy	0.73		0.41	0.76	0.47	0.55
	Sensitivity	0		0.41	0	0.21	0.2
	Specificity	1		0.58	0.93	0.82	0.79
	Subtype	–		0.83	–	0.4	0.59
Global connectivity	Accuracy	0.73				0.49	0.53
	Sensitivity	0				0.33	0.27
	Specificity	1				0.82	0.85
	Subtype	–				0.38	0.38
Regional homogeneity	Accuracy	0.55		0.34	0.71	0.49	0.5
	Sensitivity	0		0.41	0	0.21	0.2
	Specificity	0.75		0.5	0.86	0.85	0.74
	Subtype	–		0.67	–	0.4	0.52
Raw rs-fcMRI connectivity matrices	Accuracy		0.6	0.37		0.49	0.47
	Sensitivity		0.27	0.28		0.33	0.3
	Specificity		0.86	0.58		0.74	0.71
	Subtype		1	1		0.63	0.84
Nodal/global graph metrics	Accuracy		0.52	0.39	0.56		0.48
	Sensitivity		0.45	0.17	0.33		0.3
	Specificity		0.57	0.92	0.68		0.75
	Subtype		1	1	0		0.66
Power spectra	Accuracy		0.52	0.44	0.82	0.53	0.57
	Sensitivity		0.18	0.55	0.17	0.08	0.25
	Specificity		0.79	0.58	0.96	0.96	0.83
	Subtype		1	0.69	1	0.5	0.75
Freesurfer subcortical	Accuracy		0.8				0.8
	Sensitivity		0.55				0.55
	Specificity		1				1
	Subtype		1				1
Freesurfer cortical	Accuracy			0.51	0.62		0.56
	Sensitivity			0.41	0.33		0.38
	Specificity			0.75	0.71		0.73
	Subtype			1	0.5		0.77

Classifier metrics include 3-class accuracy, 2-class sensitivity/specificity, and ADHD subtype accuracy. Data for Pittsburgh and Brown sites were not available. Blank cells indicate modalities that were omitted from a particular final classifier due to feature ranking/selection predicting they were not expected to improve performance over chance.

## Discussion

In the current work, we presented our method for classifying the ADHD-200 dataset based on structural and functional neuroimaging data, feature selection with SVM-RFE, individual site- and modality-specific classifiers, and voting to assign final class labels. This approach outperformed all measures of chance-level performance, and was competitive among the other entries in the ADHD-200 Global Competition. Looking forward, this vast neuroimaging dataset provides an excellent resource for studying ADHD. Machine learning and classification tools may provide new means for exploring these data, and may help to determine which underlying neural features are related and perhaps give rise to ADHD.

### Demographics

Even without exploring the imaging data or applying more advanced ML approaches, the demographics data alone were able to provide valuable insight into ADHD classification. The usefulness of these features is clear from examining Figure 6, where prominent site, gender, and IQ effects are all visible. However, no age effect on ADHD diagnosis was present, and this was not a useful feature. The fact that the team with the best accuracy in this competition used only demographic measures raises two important points. First, demographic variables may currently provide more clinical utility than neuroimaging features, particularly for heterogenic neuropsychiatric disorders like ADHD or Autism Spectrum Disorders. Second, the composition of subject pools at different sites is an important factor for designers of future ML competitions to consider, as site-specific biases in subject inclusion may bias results to favor a demographic-only classifier.

#### Overall ADHD prevalence

The most basic observation on the training and test datasets was that there were more TD subjects than ADHD subjects. However, the prevalence of ADHD in the sample was still much higher than what would be found in a real community population. This simple fact is critical to appreciate because it lays down the general pretest probability for whatever final classifier is developed. For example, given a test subject with equivocal imaging features, we would like a classifier that does not simply assign diagnosis based on 50/50 chance, but would favor a moderately higher probability diagnosis of TD based on the higher representation of TD subjects across the training set.

In diagnostic testing terms, the lower the pretest probability of ADHD, the lower the positive predictive value of our test. After all, even if we develop a test that is 99.99% accurate at diagnosing some disease, if we know for a fact that the prevalence of the disease in the population is 0%, than *all* of the positive test results are still going to be false positive type I errors. Consequently, the barrier to developing a useful diagnostic test is higher in the real world, where the disease prevalence is typically lower than in controlled studies, and there are additional factors to consider such as cost and potential treatment risks.

#### Site

The large variation in ADHD prevalence and subtype ratios between sites is also important to explore. While these specific numbers are of course artifacts of sampling bias across the individually-designed studies that joined ADHD-200, they again highlight the importance of tuning our classifier to the variable prevalence within sub-groups in our population. Similar diversity also exists in the real world, as, perhaps, some regions of the country have a higher prevalence of a certain disease due to differing demographic, genetic, or environmental factors. In the ADHD-200 sample, these observations helped build an intuitive understanding of how our classifier should perform. We already knew that designing a classifier to resolve a behaviorally diagnosed disease like ADHD from TD, based on brain imaging data alone, would be challenging. On top of that, it seemed unlikely that imaging features would outperform the very strong baseline expectations about which subtype to expect at which site. Therefore, we decided early on that we would concentrate our effort on classifying ADHD from TD, generally, and would default to these prior expectations for assigning subtypes to the ADHD subjects.

#### Gender

ADHD is more common in males than in females, so we expected this feature would be very useful as well. Indeed, across the whole dataset, the ADHD prevalence among males was roughly twice as high as females. This substantial effect is inline with what has been observed in the general population (Morbidity and Mortality Weekly Report, 2010), and suggested that our pretest expectations about ADHD diagnosis should vary prominently based on whether a given test set subject is male or female. This effect also varied by site, which again reiterates the need to address site, and any other influential demographic subpopulations/factors, when designing clinical diagnostic aids.

#### IQ

Considering the strong relationships between cognitive measures and ADHD diagnosis that have been previously reported, it came as no surprise that the full-scale IQ measure was also a highly informative feature. For example, Kuntsi et al. (2004) observed that individuals with ADHD scored nine points lower than TD controls, and that the co-occurance of low IQ and ADHD likely has a common genetic origin. When the IQ vs. age plots in Figure 6 were examined, several findings were clear. First, considering the marginal distributions, there was no striking gender effect on age or IQ (i.e., age and IQ were relatively well-matched across genders), but there was a strong site effect on both age and IQ. At the extremes, OHSU didn't have *any* subjects older than 12 years old, and Pitt didn't have any subjects younger than 10. Similarly, OHSU females had exceptionally high IQs. Secondly, considering main effects, there was a strong correlation between lower IQ and ADHD diagnosis, but no appreciable age effect was present. Lastly, considering the joint usefulness of age and IQ for predicting diagnosis, one can see a large degree of variability between sites and genders. For example, whereas Peking subjects separate nicely based on these features, NYU subjects do not. When IQ was placed in the same lists as functional neuroimaging features for feature ranking, it repeatedly rose to the top of our ranked feature lists even when many thousands of features were present. This not only quantitatively highlights the usefulness of the IQ features, which we have previously discussed only qualitatively, but also demonstrates the effectiveness of the SVM-RFE algorithm on very large feature sets.

### Classifier design

The robustness of our feature selection approach is one of the main positive aspects to take away from our effort. The mSVM-RFE method was able to handle large feature sets (e.g., the CC400 atlas provides 79,800 unique features after duplicates and self-correlations are removed) in a reasonable amount of time, and consistently returned useful ranked lists of the top features. When examining the plots of expected generalization performance versus the number of top features used as input, it was interesting to see that the shape of these curves varied by imaging modality. For example, in Figure 3B we can see that only a few of the most highly ranked features were enough to efficiently summarize the useful morphological aspects of the data. Conversely, in Figure 3A, the rs-fcMRI correlation matrices typically gave poor performance when only a few of the top features were used, and instead required more features to be included in order to reach optimal performance. This variation suggests that the true intrinsic features of the rs-fcMRI data are more distributed network-type properties, rather than specific isolated effects. For all types of imaging modalities, adding extraneous features beyond the optimal zones caused expected performance to drop off toward chance. For many types of classifiers, this is due to the “curse of dimensionality” that arises when the amount of available data becomes sparse in a higher dimensional parameter space (Hughes, 1968). However, SVMs are actually less susceptible to this common problem since they do not require accurate models of class distributions throughout the entire multidimensional space, but rather rely on data exemplars only in the neighborhood of the decision boundary between the classes (Melgani and Bruzzone, 2004). Still, many of the neuroimaging features will be useless, and adding these will only contribute noise to the data and decrease overall classifier performance.

Interestingly, while there *are* non-linear methods for doing feature selection (e.g., wrapping the whole classifier and using the predicted generalization accuracy as the objective function), simpler linear methods have been advocated in this type of preprocessing role because of their speed and ability to reduce dimensionality with less risk of overfitting (Guyon, 2003). However, one potential limitation of this design is that it could miss features that appear useless at the linear stage, but are actually highly useful when used with a non-linear classifier (e.g., imagine a U- or doughnut-shaped decision boundary). While this is an interesting theoretical consideration, in practice many biological and imaging based relationships—although non-linear—are still generally monotonic and therefore effectively identified by this type of linear feature selection method. Indeed, the winning entries in large ML competitions across diverse datasets often use the simplest of feature selection approaches—including univariate correlation based ranking, or principal component analysis (Guyon et al., 2005).

### Interpretation of neuroimaging features

A reasonable criticism of ML in neuroscience is that it is entirely possible to develop algorithmic classifiers that distinguish states or pathologies with high accuracy based on features that do little to inform basic understanding. Neuroimaging data display a nearly unbounded set of possible features. Ideally, the dimensions used to describe the data are themselves interpretable. In such cases, the class boundaries calculated by the classifier in its training phase form a “hidden layer” that can also be informative. Our method for optimal feature subset selection pared down the vast number of neuroimaging features to a more tractable, parsimonious number—typically on the order of 10 to several hundred features.

Although the total number and specific informative features used for generating a categorical vote varied across sites, a number of highly ranked features were consistent across sites. From the subcortical features, regional voxel intensity means in left caudate and right thalamus were ranked highly, consistent with previous structural studies in ADHD (Ellison-Wright et al., 2008; Ivanov et al., 2010). Even highly ranked morphological features of non-gray matter regions, such as the volume of left inferior lateral ventricle volume, have previous precedence in the literature (Verkhliutov et al., 2009). Structural cortical measures that were highly diagnostic were located primarily in frontal, temporal, and cingulate regions, again demonstrating partial correspondence with previous reports of altered frontal circuitry in the context of ADHD (Shaw et al., 2006; Qiu et al., 2011). Importantly, our detection of structural differences in the dorsolateral PFC, a region critical for attentional control, aligns with the primary affected cognitive process in ADHD. For the functional connectivity matrices we found that a variety of connections distributed across the brain were informative, which is not surprising considering that behavioral disorders affect aspects of behavior and multiple brain networks. However, we did find more informative features were more lateralized to the right hemisphere and graph theoretical nodal features were more specific to frontal and temporal regions. Features from the voxelwise measures of regional homogeneity and global connectivity also pointed toward multiple brain regions including regions known to be part of the default mode and attention networks. In both voxelwise results, it is also interesting to note that the voxels with the highest group-average global connectivity or regional homogeneity measure were not necessarily the same voxels with the highest feature rankings.

### Classification across sites

Although the ADHD-200 multisite dataset has been utilized already in the neuroscience community to further our models of ADHD and motivation (Tomasi and Volkow, 2012), protocol variations between sites led to large inter-site differences in measurement. This highlights the need for consistency in procedures to make data sharing efforts most effective, which will allow for an enhanced ability to replicate results (or compare differences) between different analyses. For example, the IQ scores across the ADHD-200 sample were derived from different raters and different test instruments, depending on site, and the NI site didn't provide any IQ data at all. This posed the challenge of how to best utilize this useful feature at most sites, but not all, and also while accommodating potential site effects. Even more extreme, the Pitt site only included TD subjects in its training data, and the Brown site did not provide *any* training data. The challenge with these sites was how to design an *across*-site classifier, without inadvertently biasing the predictions due to nuisance site effects (e.g., scanner-specific signal biases, varying baseline ADHD prevalence, etc.).

As described, our general approach to address all of these issues was to use a series of site-specific classifiers and, where across-site classifiers *were* required (Pitt, Brown), a feature- and site-wise bias correction. We chose this site-specific approach after initial analyses suggested that top features were quite different between sites and classifiers trained across sites performed worse than within-site classifiers. While this approach was useful, in effect it largely forgoes the potential benefits of a true multisite study, and instead may be better described as a meta-analysis of ADHD classification. It is likely that cross-site uniformity in multisite studies should improve classification accuracy, as it is easy for uncontrolled parameters to swamp out biological signal. Conversely, for a biomarker to be clinically relevant, it should also be consistent across sites and robust to variations in imaging parameters. This suggests that there is currently not a strong neuroimaging signal or biomarker for ADHD, or at least that any signal is smaller than the variability introduced by including multiple sites with different scanning parameters and samples that are unmatched for demographics.

### Performance on test data

The most surprising result to come out of the ADHD-200 competition was that, although imaging features were moderately useful for classifying ADHD from TD subjects, including these features failed to provide any additional benefit over using demographic features alone. There were 195 possible points (two of the 197 original test subjects were excluded), which would have required correctly predicting all ADHD subjects as well as all ADHD subtypes. The winning *imaging*-based classifier scored 119 points. For reference, our approach scored 110.5 points. However, the best *overall* score of 124 points was reached by ignoring the imaging features completely and relying solely on the demographics information. Interestingly, this is close to the 127 points we predicted early on based on our own analysis of a subset of the demographics features in the training set (Section “Expected classifier performance”). Nevertheless, we had decided to continue investigating the imaging features for two reasons: (1) those were the main focus of the ADHD-200 project, and (2) we reasoned the imaging features would be more generalizable, as we suspected some of the demographics effects were simply artifacts of study design. Still, this finding is a good reminder that when we see claims like “Feature X is useful for classifying disease Y,” we should ask the question, “Relative to what baseline?” In this case, the imaging features *were* useful—just not above and beyond the much simpler demographic information. Similarly, it is also important to consider the performance we could have expected based on chance alone. Based on hierarchical coin flipping, the chance level was 39% (corresponding to 86.5 points). It is also useful to examine the “no-information rate,” which is the performance we would attain by predicting that all of the test set cases belong to the most common training set class. Seeing as TD was the most common overall diagnosis in the training set, one would have achieved 55% accuracy (corresponding to 108 points) just by predicting all of the test subjects as TD. With respect to the performance of the imaging-based classifiers, this number is just as striking as the demographics-only performance. It suggests that around half of the imaging-based competition entries would have performed better—and certainly expended less effort—by simply classifying everyone as TD. This relatively modest performance challenges us to question whether the imaging features present in the data are the “right” ones from which to reach a clinical finding. It is possible that the abnormalities in morphology and resting-state signal fluctuations considered at present are associated only weakly with the disease, and that a different set of features or imaging modality might be needed for neuroimaging to have a large impact on the clinical management of ADHD. Considering the large individual variability that accompanies even real group effects, and the relatively low prevalence of many diseases in community populations (compared to research studies), this also highlights the more general challenge of attempting to use neuroimaging features for true *diagnostic* classification of behaviorally-diagnosed neurological syndromes like ADHD. While this is still an excellent long-term goal, imaging-based classifiers of ADHD and other such disorders show their biggest short-term promise in populations where the pretest probability is high or diagnosis is already assumed. For example, in a scientific context toward further understanding the neurobiological basis of the disorder, these techniques can be used to map regions of the brain that are most useful for classification (Uddin et al., 2011), thereby providing a complementary tool to standard univariate hypothesis testing. Similarly, in a clinical context, they may be more useful in predicting diagnostic subtypes among individuals who have already been screened from the population at large, or for predicting later treatment response and prognosis.

### Future directions and conclusion

Overall, this competition provided one of the largest and potentially most valuable public neuroimaging resources for studying any neurodevelopmental disorder, and the largest ML collaboration in the medical community to date. This was only possible through the strong cooperation between the organizing sites, together with their progressive open-access philosophy toward data sharing. Even more exciting is the response that the community has had in building on this foundation, including the preprocessed versions of the original fMRI dataset that were contributed back to the community by the Neuro Bureau. These types of efforts lower the entry barrier to the field, and promote a collaborative synergism that accelerates research discovery.

Although imaging features have showed only modest classification performance thus far, improved classification accuracy will likely come with advances in imaging acquisition and modeling methods, standardization of protocols across sites, larger sample sizes, as well as a better understanding of genetic factors influencing these circuits. Nevertheless, our optimism for the future must be tempered by realistic expectations for what neuroimaging data can and cannot do for us (Logothetis, 2008), and the appreciation that there will always be a large degree of heterogeneity due to normal individual variation in cognitive profiles and neural circuitry (Fair et al., 2012). Ultimately, the ADHD-200 initiative is leading the way toward a productive new era of neuroscience collaboration, but it still remains to be seen if and when our growing understanding of the neural basis of ADHD will eventually begin to lead to improved clinical outcomes over the current standard of care.

### Conflict of interest statement

The authors declare that the research was conducted in the absence of any commercial or financial relationships that could be construed as a potential conflict of interest.
